# Addressing the Barrier of Physical Access to Oncology Care in Sub-Saharan Africa (SSA) Through Establishment of Short-Stay Homes for Cancer Patients: A Case of International Cancer Institute, Child and Family Wellness Center in Eldoret, Kenya

**DOI:** 10.1200/GO.22.16000

**Published:** 2022-05-05

**Authors:** Gloria Kitur, Florence Mbandar, David Muyodi, Chite Asirwa

**Affiliations:** ^1^ International Cancer Institute, Eldoret, Kenya; ^2^ Creation Hive, Eldoret, Kenya

## Abstract

Geographical distance to access services and lack of decentralized diagnostic and treatment facilities have been identified as barriers to positive treatment outcomes among cancer patients in Sub-Saharan Africa who have to undergo financial and logistical challenges. Potential approaches to reducing this barrier include providing transport/travel allowance to patients from far flung regions, decentralizing cancer care services or providing patient housing close to cancer treatment facilities.

**METHODS:**

The International Cancer Institute Child and Family Wellness Center was established in June 2020 as a half-way home for patients undergoing treatment at the ICI Cancer Care and Research Clinic (ICI_CRC). The center aims to provide safe accommodation for patients having challenges to travel to and from their homes for diagnostic work-up and treatment.

**RESULTS:**

Between June 2020 and October 2021, a total of 101 admissions were made to the ICI Child and Family Wellness Center, of these 76% were female while 24% were male patients. A majority of the patients were first time admissions while 22% of them were admitted more than once; 69% of them were accompanied by a caregiver. The longest duration of stay was 18 days while the shortest was 1 day depending on the reason for admission; the most common being treatment sessions ending late in the day or treatment sessions taking more than one day. The patients admitted were drawn from 15 different counties in Kenya.

**CONCLUSION:**

Physical inaccessibility to cancer care services in SSA greatly affects patient compliance with treatment and follow-up. Patients who live far from specialist care hospitals are more likely to have an advanced disease at diagnosis, inappropriate treatment, a worse prognosis and poorer quality of life compared to those living closer to hospitals. In addition to decentralization of cancer care, provision of accommodation for cancer patients close to hospitals can greatly improve compliance to treatment resulting in positive treatment outcomes.

